# Identification of novel targets and pathways to distinguish suicide dependent or independent on depression diagnosis

**DOI:** 10.1038/s41598-023-29101-1

**Published:** 2023-02-13

**Authors:** Siqi Peng, Yalan Zhou, Lan Xiong, Qingzhong Wang

**Affiliations:** 1 Institute of Chinese Materia Medica, Shanghai University of Traditional Chinese Medicine, Shanghai, 201203 China; 2 Montreal Neurological Institute and Hospital, McGill University, Montreal, QC Canada

**Keywords:** Data mining, Depression

## Abstract

In recent years, postmortem brain studies have revealed that some molecular, cellular, and circuit changes associated with suicide, have an independent or additive effect on depression. The aim of the present study is to identify potential phenotypic, tissue, and sex-specific novel targets and pathways to distinguish depression or suicide from major depressive disorder (MDD) comorbid with suicide. The mRNA expression profiling datasets from two previous independent postmortem brain studies of suicide and depression (GSE102556 and GSE101521) were retrieved from the GEO database. Machine learning analysis was used to differentiate three regrouped gene expression profiles, i.e., MDD with suicide, MDD without suicide, and suicide without depression. Weighted correlation network analysis (WGCNA) was further conducted to identify the key modules and hub genes significantly associated with each of these three sub-phenotypes. *TissueEnrich* approaches were used to find the essential brain tissues and the difference of tissue enriched genes between depression with or without suicide. Dysregulated gene expression cross two variables, including phenotypes and tissues, were determined by global analysis with *Vegan*. RRHO analysis was applied to examine the difference in global expression pattern between male and female groups. Using the optimized machine learning model, several ncRNAs and mRNAs with higher AUC and *MeanDecreaseGini*, including *GCNT1P1* and *AC092745.1, *etc., were identified as potential molecular targets to distinguish suicide with, or without MDD and depression without suicide. WGCNA analysis identified some key modules significantly associated with these three phenotypes, and the gene biological functions of the key modules mainly relate to ncRNA and miRNA processing, as well as oxidoreductase and dehydrogenase activity. Hub genes such as *RP11-349A22.5*, *C20orf196*, *MAPK8IP3* and *RP11-697N18.2* were found in these key modules. *TissueEnrich* analysis showed that nucleus accumbens and subiculum were significantly changed among the 6 brain regions studied. Global analysis with *Vegan* and RRHO identified *PRS26*, *ARNT* and *SYN3* as the most significantly differentially expressed genes across phenotype and tissues, and there was little overlap between the male and female groups. In this study, we have identified novel gene targets, as well as annotated functions of co-expression patterns and hub genes that are significantly distinctive between depression with suicide, depression without suicide, and suicide without depression. Moreover, global analysis across three phenotypes and tissues confirmed the evidence of sex difference in mood disorders.

## Introduction

Major depressive disorder (MDD), a common mental illness, has become the leading cause of disability and disease burden worldwide^1^. MDD is highly associated with suicide. On the one hand, approximately 15% of MDD patients have their lifetime risk for suicide^2^; on the other hand, nearly 60% of individuals who died by suicide had a history of mood disorders^3^. Historical and recent data have shown that over 85% individuals who died by suicide had psychiatric disorders, including affective disorders, particularly in females^4–6^. Some MDD risk factors, including childhood adversity, stress, hopelessness, and aggressivity, may contribute to the activation of suicide ideation across a wide range of the lifespan^7–10^. Although some previous studies on the etiology of suicide have suggested that some neurobiological abnormalities of suicide ideation and suicidal behavior are independent of psychiatric disorders, extensive postmortem brain studies have revealed that some changes at the molecular, cellular and circuit levels that may trigger suicide, have both independent and additive effect on depression through different pathways and brain circuitries^11–14^.

To date, large-scale genome wide association studies (GWAS) of MDD have produced some consistent genetic risk factors that contribute to susceptibility to depression^15–19^. For example, a meta-analysis of 3 largest MDD GWASs has identified 102 independent variants, 269 genes, and 15 gene sets, which have shown genome-wide significant association with depression^20^. The most recent meta-analysis of MDD GWAS has included 366,434 cases and 847,433 controls with both Caucasian and African background, has uncovered 233 independent significant SNPs at 183 genomic risk loci^21^. Subsequently, the integration of GWAS results and transcriptional data was further conducted in the context of blood and postmortem brain tissues of depression. However less such genome-wide significant results were found in suicide^22–25^. From the view of multi-omics on postmortem brain tissues, transcriptomic and epigenetic changes in depression and suicide have been recapitulated using postmortem brain tissue as study materials that directly reflect changes relevant to the neurobiology of suicide and psychiatric disorders^26–30^. By examining some potential functional candidate genes, abnormalities in the serotonergic, glutamatergic, and GABAergic systems, as well as the hypothalamic–pituitary–adrenal axis for stress response, and the inflammatory pathways, have been detected in depressed or suicidal brain tissue^31–34^. More recent transcriptomic studies on different brain regions of depressed and suicidal individuals have not only confirmed the previous findings on GABA- and glutamate-related genes, etc., but also have discovered some novel genes and important signaling pathways^35–42^. Among these transcriptomic studies, a large-scale transcriptomic study of different brain tissues from depressed patients, who died from suicide, first directly explored the molecular mechanism of sexual dimorphism in MDD. This study has surprisingly shown that very little overlapping of differentially expressed genes was shared between males and females, both in depressed humans and with stressed mice^43^. Meanwhile, the sex-specific hub genes from the gene co-expression patterns (modules), e.g., *DUSP6* and *EMX1*, were involved to promote stress vulnerability in female and male mice, separately^43^. Another transcriptome study of depression and suicide examined the genome wide exon gene and microRNA expression profile in the dorsal lateral prefrontal cortex of different types of postmortem brains, including non-psychiatric controls, MDD with suicide, and MDD non-suicides. The results of mRNA-seq GO analysis demonstrated that the genes involved microglial, endothelial, and glial cell DNA-dependent ATPase activity contributed to MDD and suicide^44^. Considering that a significant fraction of postmortem human brain donors with depression died of suicide, here we have performed a series of bioinformatics analyses with these two high-quality datasets to identify the novel targets of suicide, which are distinctive from the main pathways for MDD.

## Subjects, data and methods

### Postmortem mRNA expression data and reclassification of sub-phenotypic groups

Two previously reported postmortem mRNA expression profiling datasets for MDD studies were retrieved from the GEO database (GSE102556 and GSE101521). Besides of the corresponding gene expression data, we also obtained demographic data, as well as suicide information from each subject. We summarized the demographics and clinical characteristics, and re-grouped the subjects, based information from concomitant MDD and suicide information from these two datasets (Supplementary Table 1).

The GSE102556 study (PMID: 28825715) included 48 human subjects (age: 47 ± 15 years, F:M = 22:26); each subjects had 6 postmortem brain regions, including Anterior insula (Ant), orbitofrontal (BA11), cingulate gyrus 25 (BA25), dorsolateral prefrontal cortex (BA8/9), nucleus accumbens (Nac) and subiculum (Subic). Based on the occurrence of suicide and MDD diagnosis, the subjects from the GSE102556 study, i.e., the Suicide Group, were reclassified into three groups: suicide with MDD (S + D_1, n = 26, age: 45 ± 14 years, F:M = 13:13), suicide without MDD (S-D_1, n = 11, age: 48 ± 17 years, F:M = 5:6), and non-suicide without MDD (CTR_1, n = 11, age: 57 ± 16 years, F:M = 4:7).

The GSE101521 study (PMID: 27528462), i.e., the Depression Group, included 59 subjects (age 49 ± 21 years, F:M = 17:42) with one postmortem brain region of dorsolateral prefrontal cortex (BA9), which were reclassified as MDD with suicide (D + S_2, n = 21, age: 52 ± 22 years, F:M = 8:13), MDD without suicide (D-S_2, n = 9, age: 58 ± 16 years, F:M = 3:6), and control (CTR_2, n = 29, age: 44 ± 21 years, F:M = 6:23). The clinical and demographic information of these participants in this study was collected by experienced psychiatrists and care-providers; and the clinical diagnostic criteria for the disease were referred to DSM-IV and SCID-1D.

The gene expression data from one overlapped brain region, i.e., the dorsolateral prefrontal cortex (BA9), from these two datasets were further merged and normalized according to the standard protocol; and the batch effect was removed by the *limma* package (v3.36.5)^45^. An aligned and normalized gene expression data-frame was generated for each subject. Principal variance component analysis with *ExpressionNormalizationWorkflow* was performed to estimate the data structural variances, and the significant expression principal components (PCs), adjusted for known covariates, including sex, age, postmortem interval (PMI), RNA integrity number (RIN)^46^.

### Differential gene expression analyses

To find out the characteristic gene expressions as candidate mRNA for different sub-phenotypes, machine learning analyses were performed for the merged gene expression dataset from the BA9 region. First, the normalized TPM (transcripts per million) matrices were proceeded using linear fit in the *limma* package to perform differential expression analyses of three comparisons, including S + D_vs_CTR, S-D_vs_CTR, and D-S_vs_CTR. Genes with a false discovery rate (FDR) less than 0.05 were defined as differentially expressed genes (DEGs), as previously reported^47^. The overlapping DEGs between each comparison were plotted as *venn diagram*.

### Machine learning analyses

Then, the genes that were specifically enriched with one sub-phenotype were considered as candidate molecules; and the machine learning model was built with *randomforest* algorithm as previously reported^48^. During the training and testing stage, all subjects were divided into two classes using the *createDataPartition* function, about 80% of the participants were assigned to the training part, while the remaining 20% of the subjects were assigned to the testing part. Meanwhile, we optimized the two important parameters of *mtry* and *ntree* of the machine learning model to obtain the smallest estimation error rate so that it could have discriminate between classifiers. Among these two parameters, *mtry* represent the number of variables randomly sampled when building a decision tree branch in random forest modeling; and the optimal value of *mtry* parameter reflects as the lowest prediction error rate of the model. While *ntree* stands for the number of decision trees. In general, the lower ntree value will produce higher error rate while the higher *ntree* value will increase the complexity of model and reduce the prediction efficiency. Therefore, we first determined the optimal "*mtry*" parameter and the number of *ntrees*, based on the above candidate predictors as variables and *tuneRF* function. Subsequently, tenfold cross-validation was performed to avoid overfitting the model while determining the number of DEGs for the optimal classifier. Here, we introduced the Gini index as an evaluation index for the importance of the features (DEGs) the value of which was proportional to the number of sample splits. Usually, the higher value of the Gini index for a feature (DEGs), the more important it is. The genes having higher Gini Index were selected to input the random forest model and to calculate the area under the curve (AUC) values using the true class labels of half of the tested samples.

### Weighted gene co-expression network analysis (WGCNA)

In order to further identify distinctive gene networks and pathways associated with three subphenotypes, we performed a weighted gene co-expression network analysis (WGCNA). First, a standard linear two-way analysis of variance was performed with *aov* function independently on the data for each gene^49,50^. The effects examined for each gene were difference among three phenotypic groups. Known covariates (e.g., sex, age, PMI, RIN) were adjusted with *glm* function for each gene, and significant genes in the two datasets were used for the following WGCNA analysis. WGCNA was conducted to identify key modules and hub genes significantly associated with a particular phenotype. Following the WGCNA tutorials and our previously reported studies, the soft-threshold β was selected using the *pickSoftThreshold* function based on the uncertain scale-free topology^51–53^. First, the similarity matrix was obtained by calculating the correlation coefficient between genes and then converted into an adjacency matrix with the function *pickSoftThreshold*, which can determine the best soft threshold/value and average degree of connectivity on the conditions of scale-free network construction. Here, we chose the value equal to 7 as the soft threshold at this point of the fitting index just reaches 0.85. Then, we transformed the adjacency matrix into the topological overlap matrix by means of the function *TOMsimilarity*. From its dissimilarity of topological overlap matrix, genes with similar expression patterns were divided into the same module labeled with a certain color, where minModuleSize of each module can be a set 30 genes as default number, and the genes in the gray module are not involved in subsequent research. Subsequently, we calculated the Moduleeigengene (ME) of each module which was defined as the first principal component of all gene expression data in the specific module and represented the overall level of gene expression. The spearman correlation coefficient from ME and phenotype trait association analysis was used to analyze the correlation between the module and phenotypic characteristics. Then, we selected the key modules with the largest correlation coefficients and the smallest p values by observing the results in the module feature map. Finally, *chooseTopHubInEachModule* function was utilized to identify the hub genes in the associated modules, which were visualized in the form of network graphs plotted with *igraph* package. To annotate the biological functions of key modules, *clusterProfile* package was used to enrich the genes in the modules into biological pathways and pathways.

### Tissue-specific and sex-specific gene expression for suicide

We further explored the GSE102556 dataset based on re-organized dataframe based on suicide with and without MDD sub-phenotypes for tissue-enrichment and sex-specific effects on gene expression. First, we analyzed the transcript ID expression changes across six types of brain tissues using the *aov* function, which represents the similarity and difference between different tissues. To be specific, it will be spitted into three different dataframes, corresponding to 3 phenotype groups. ANOVA was performed to test for differences among 6 independent tissue groups for each gene. The p value of ANOVA analysis was corrected by cofounding factors. This cross-tissue analysis was individually performed in three phenotypic groups (S+D_1, S−D_1 and CTR_1). Also, the significant level of each gene will be acquired as differentially expressed genes (DEGs), which showed the variance among the six regions. To further explore the enriched tissues of these significant genes, we conducted the tissue-specific gene enrichment analysis using the *TissueEnrich* package^54,55^. Referred as the *TissueEnrich* handbook, the dataframe containing expression information (rows as genes, columns as tissues) was input into *SummarizedExperiment* function. The *teGeneRetrieval* function was used to calculate tissue-specific gene enrichment. According to *TissueEnrich* tutorials, the default threshold can be optional changes to alter the degree of tissue specificity of the gene. In current study, we set the threshold as 4-fold changes, which means genes from tissue enhanced groups which reach at least 4-fold compared to the average levels in all other tissues, are considered brain tissue-specific genes in suicide with depression and suicide without depression groups.

In order to investigate the global difference between the transcriptional signature of whole brain tissues under the two-layers of phenotypes and tissues, Analysis of Similarity (ANOSIM) was performed with the function *anoism* from the *Vegan* package to simultaneously detect the dysregulation of gene expression in the multidimensional scaling across three phenotypes and six tissues (cross phenotype and tissue analysis)^56^. As a non-parametric statistical test, ANOSIM has been widely used in the field of ecology; and the objective of ANOSIM mainly operates on a ranked dissimilarity matrix instead of raw data. First, one dataframe was produced containing the mRNA expression level for the total of samples from three groups and 6 tissues. Then, the function *vegdist* will produce a suitable dissimilarity matrix from raw dataframe; and *anosim* function directly operates on dissimilarity matrix with permutation correction. If the entire brains from the three phenotype groups do differ, then the difference between the three groups should be greater than the difference within the group. The heteromorphic statistic R (R = (rB − rW)/(N(N − 1)/4)) is calculated on the basis of difference in mean ranking between groups (rB) and within groups (rW). The statistical significance of the observed R was calculated by permuting the grouping vector to obtain the empirical distribution of R under the null model. We then divided all subjects into male and female groups and performed ANOSIM analysis in each of the male and female groups, as well as calculated the global significance level across phenotypes and tissues in each of the male and female groups.

The extent of overlap of dysregulated genes highlighted in male and female subjects were examined by performing unbiased RRHO (v1.26.0), as described previously^57–60^. With a threshold-free method, RRHO analysis mainly estimate overlap between two ranked lists of genes. To perform RRHO analysis, the two gene sets were firstly ranked at the genome-wide scale according to their − log10 (P value) and the direction of change revealed by differential expression analyses. Secondly, a series of hypergeometric p values were calculated for each gene by sliding the rank threshold to examine the significance of overlapping genes above the expected threshold at each ranking site. Lastly, the hypergeometric p values of genes were plotted on a heatmap.

### Ethical approval

The Ethics Committee of the Shanghai University of Traditional Chinese Medicines waived the ethics approval and consent for the collection, analysis and publication of the transcriptional data for this study.

## Results

### Identification of DEGs, co-expression patterns, and hub genes for suicide with depression, suicide without depression and depression without suicide

**Figure 1:**
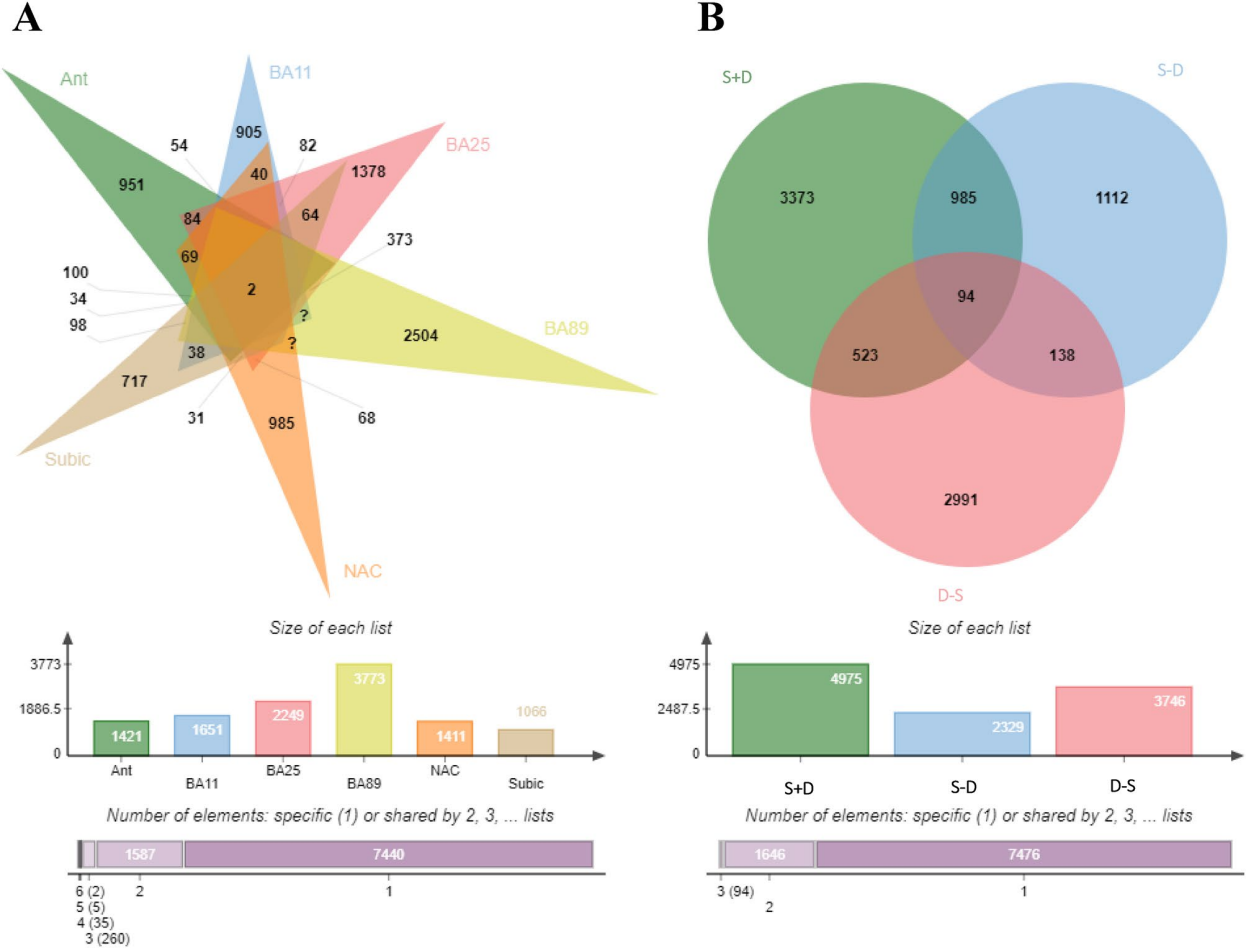
Similarity and overlapping in the DEGs between different tissues and diseases. (**A**) DEGs from the cross-phenotype analysis. Different colors represent different tissues, overlapping parts represent genes present in all tissues, parts of not overlap represent DEGs that are specific to the tissues, and numbers indicate the number of DEGs in the corresponding tissue. (**B**) Venn diagram of the different phenotypes. Colors represent each phenotype, overlapping parts represent shared genes among phenotypes, parts of not overlapping represent DEGs that are specific to the diseases, and numbers indicate DEGs numbers of different phenotypes.

The significant p value of each transcript ID is sequentially calculated with *aov* function to show the changes of dysregulated expression genes across three phenotypic groups (Suicide Group: CTR_1, S+D_1, and S−D_1; Depression Group: CTR_2, D−S_2, D+S_2). This cross-phenotype analysis was separately conducted in the six brain regions in Suicide Group and one region in Depression Group. Then, the *ExpressionNormalizationWorkflow* was utilized to determine the variance of the principal components of the DEGs expression data that were impacted by demographic and other confounding factors. The results suggested sex, age, alcohol abuse and RNA integrity had a significant effect on the principal components (p < 0.05). Therefore, these demographic factors were used as confounding factors to correct for any significant difference. After correction with logistic regression analysis, the total number of 2467 DEGs were screened in the Depression Group (Supplementary Table 2; Supplementary Fig. 1). Regarding the number of DEGs in the six tissues in the Suicide Group, the most DEGs (n = 3773) were identified in the dorsolateral prefrontal cortex while the least DEGs (n = 1066) in the subiculum (Supplementary Table 3; Supplementary Fig. 2). Some genes, such as *DUSP6* and mitogen-activated protein kinase genes, have been previously reported in the depression and suicide study while more novel genes were found. In general, the changes trend for most genes were observed to be upregulated in the Suicide Group (Supplementary Table 3). The similarities and differences genes of the 6 regions were shown in *venn diagram* (Fig. 1A). After examining gene expression in the 6 tissues, two transcripts containing *RP11-459O16* belonging to the lncRNA family and *CH25H* coding with cholesterol 25-hydroxylase have significant changes overlapping in the 6 regions, suggesting that these two genes may play a contributory role in the connectivity of different brain tissues.

**Figure 2:**
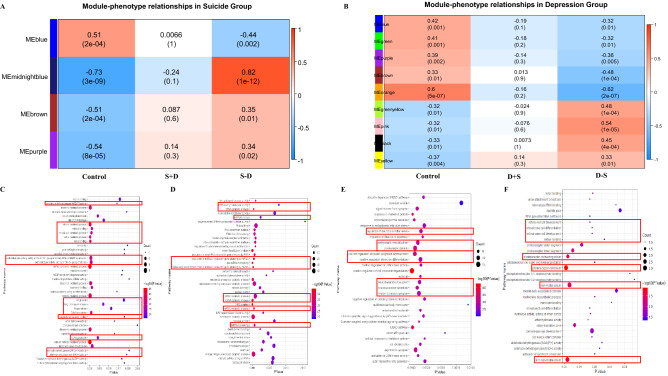
Module-trait relationship analysis with WGCNA and the KEGG pathway analysis for the correlated modules. (**A**) Module-phenotype relationship for the assigned module and different phenotypes from Suicide Group. Values in the figure indicate the correlation coefficient between module and phenotypes. The midnightblue module was positively correlated with suicide without MDD (r = 0.82, p value = 1E−12) while the blue module was negatively correlated with suicide without MDD (r = − 0.44, p value = 0.022). (**B**) Module-phenotype relationship in the Depression Group. The two modules including the orange and pink module were the most significantly associated with MDD without suicide (orange module: r = − 0.62, p value = 2E−7; pink module: r = 0.54, p value = 1E−5). (**C**) The enriched GO functions of midnightblue module from Suicide Group. (**D**) The overrepresented GO functions of the blue module from Suicide Group. (**E**) Gene ontology analysis of the pink module from Depression Group. (**F**) Gene ontology analysis of the orange module from Depression Group.

To further investigate the co-expression genes as a module significantly associated with certain phenotype, WGCNA analysis was performed with the differential expression genes in the dorsolateral prefrontal cortex regions from these two groups. As described in the methods, 4 modules in the Suicide Group (S+D_1, S–D_1 and CTR_1) and 9 modules in the Depression Group (D+S_2, D–S_2 and CTR_2) were assigned. Subsequent analysis showed that each module was correlated with different phenotypes. Here, we focused on the modules having significant association with the phenotype of depression with suicide and suicide without depression. For example, the midnightblue module that was classified as the top positive module in the Suicide Group, showed a significant association with suicide without depression (p value = 1E−12) (Fig. 2A). Interestingly, the top significant GO annotated functions mainly relate to the activity of different types of oxidoreductases and dehydrogenase, including testosterone-, aldehyde-, and retino, etc., as well as glucocorticoid receptor binding and Golgi terms (Fig. 2C). On the other hand, the blue module in the Suicide Group, which is the top negatively correlated with suicide without depression, is mainly involved in the ncRNA, miRNA and dsRNA processing activity (Fig. 2A,D). For the Depression Group, the pink module of biological functions which focuses on the biological functions of postsynaptic synapse, has a significant negative association with depression without suicide (Fig. 2B,E). In contrast, orange module, as positive module for depression without suicide, mainly mediates into the signaling pathway for primary cilia and retinal sensitivity (Fig. 2B,F). Key hub genes were calculated based on the connectivity level of the co-expression network of each module (Supplementary Table 4). *RP11-349A22.5* in the blue module and *C20orf196* in the midnightblue module were identified as hub gene having high connectivity with nodes in a module and these two hub genes have been shown to have a key function in differentiating suicide without depression while *MAPK8IP3* in the pink module and *RP11-697N18.2* in the orange module were found to be hub genes in the module significantly associated with depression without suicide (Supplementary Fig. 3).

### Screening of important molecules as candidate targets for three sub-phenotypes

To maximally identify the potential targets for depression with suicide, we merged the subjects with depression with suicide shared these two datasets to enlarge the sample size. Also, the further PCA analysis found no stratification of cohort structure (Supplementary Fig. 4). Three comparisons, including suicide with depression and control, suicide without depression and control, depression without suicide and control, were used to conduct the comparison and contrast analysis (Fig. 1B). The total number of 3373, 1112 and 2991 DEGs were specifically assigned in the not overlapping section with suicide with depression, suicide without depression and depression without suicide, respectively (Fig. 1B; Supplementary Table 5). Then, the expression level of these specific DEGs was used to establish the machine learning model while the subjects were classified into two classes (disease state and healthy control). As described in the methods, the parameters were optimized to ensure that the model had the lowest prediction error rate. Finally, the *MeanDecreaseGini* of *GCNT1P1* and *AC092745.12* ranked as the top important candidate genes for suicide with depression (AUC = 0.90). Meanwhile, lncRNA *AC010084.1* and *RP11-1C8.7* had the highest Gini index in the S–D group, suggesting the possible link of suicide without MDD with these lncRNAs (AUC = 0.86). For depression without suicide, the *MPOL1* and *SLC2A13* gene had the highest Gini index score and may be the candidate molecules (Table 1; Supplementary Fig. 5).

**Table 1:** The genes with high MeanDecreaseGini as variables found in the machine learning analysis.

Groups	ID	Gene	Description	Biotype	MeanDecreaseAccuracy	MeanDecreaseGini	p value (suicide-group)	p value (depression-group)	GTEx expression analysis
S+D	ENSG00000236474	GCNT1P1	Pseudogene	Pseudogene	1.53	0.25	0.001	0.05	+
	ENSG00000221307	AC092745.1	AC092745.1	miRNA	1.18	0.22	0.48	0.02	+
	ENSG00000258879	RP11-713N11.5	RP11-713N11.5	LincRNA	1.99	0.22	0.68	0.007	
	ENSG00000227773	ASH1L-IT1	ASH1 like histone lysine methyltransferase	Sense_intronic	2.45	0.22	0.13	0.001	+
	ENSG00000230320	BEND7P1	BEN domain containing 7 pseudogene 1	Pseudogene	2.36	0.21	0.001	0.66	
	ENSG00000179044	EXOC3L1	Exocyst complex component 3 like 1	Protein_coding	2.17	0.20	0.35	0.001	
	ENSG00000124003	MOGAT1	Monoacylglycerol O-acyltransferase 1	Protein_coding	1.52	0.20	0.14	0.002	
	ENSG00000252023	RNU6-581P	RNA, U6 small nuclear 581, pseudogene	snRNA	1.50	0.19	0.004	0.66	+
	ENSG00000257880	RP11-769N19.2	Novel transcript, sense intronic to FAM19A2	Sense_intronic	2.19	0.19	0.0003	0.50	
	ENSG00000242986	RPL21P99	Ribosomal protein L21 pseudogene 99	Pseudogene	1.88	0.18	0.002	0.72	
S–D	ENSG00000229308	AC010084.1	AC010084.1	lncRNA	2.71	0.12	0.02	–	
	ENSG00000271830	RP11-1C8.7	RP11-1C8.7	lncRNA	1.87	0.11	0.002	–	+
	ENSG00000165379	LRFN5	Leucine rich repeat and fibronectin type III domain containing 5	Protein_coding	1.21	0.11	0.04	–	+
	ENSG00000263990	CTC-542B22.2	CTC-542B22.2	lncRNA	0.11	0.11	0.04	–	+
	ENSG00000188133	TMEM215	Transmembrane protein 215	Protein_coding	1.18	0.09	0.009	–	+
	ENSG00000262188	LINC01978	Long intergenic non-protein coding RNA 1978	lncRNA	1.27	0.09	0.02	–	+
	ENSG00000164466	SFXN1	Sideroflexin 1	Protein_coding	1.75	0.09	0.01	–	
	ENSG00000134917	ADAMTS8	ADAM metallopeptidase with thrombospondin type 1 motif 8	Protein_coding	1.20	0.08	0.003	–	
	ENSG00000253459	AL139099.1	Full-length cDNA clone CS0DK012YO09 of HeLa cells of Homo sapiens; Uncharacterized protein	Protein_coding	0.50	0.08	0.03	–	
	ENSG00000168995	SIGLEC7	Sialic acid binding Ig like lectin 7	Protein_coding	1.17	0.07	0.009	–	
D–S	ENSG00000151338	MIPOL1	Mirror-image polydactyly 1	Protein_coding	1.38	0.15	–	0.01	
	ENSG00000248865	SLC2A13	Solute Carrier Family 2 Member 13	Protein_coding	− 0.08	0.13	–	0.001	
	ENSG00000175356	SCUBE2	Signal peptide, CUB domain and EGF like domain containing 2	Protein_coding	2.04	0.12	–	0.01	
	ENSG00000249021	AC008549.2	AC008549.2	LncRNA	1.23	0.12	–	0.009	
	ENSG00000105968	H2AZ2	H2A.Z variant histone 2	Protein_coding	2.23	0.12	–	0.04	+
	ENSG00000155749	FLACC1	Flagellum associated containing coiled-coil domains 1	Protein_coding	1.56	0.12	–	0.002	
	ENSG00000171703	TCEA2	Transcription elongation factor A2	Protein_coding	0.01	0.11	–	2.10E−05	+
	ENSG00000238577	snoU13	Small nucleolar RNA U13	SnoRNA	1.67	0.11	–	0.002	
	ENSG00000183798	EMILIN3	Elastin microfibril interfacer 3	Protein_coding	0.09	0.11	–	7.91E−05	
	ENSG00000188681	TEKT4P2	Tektin 4 pseudogene 2	Pseudogene	1.71	0.10	–	0.002	

### Identification of tissue-specific genes in suicide group

To examine some genes enriched in specific brain regions, cross-tissue analysis was performed calculate the changes level of all transcripts in the group of control, suicide with depression, and suicide without depression, respectively (Supplementary Table 6; Supplementary Fig. 6). Based on these dysregulated genes from the cross-tissue analysis, the brain tissue specific genes were screened out by means of *TissueEnrich* package. The results of *TissueEnrich* demonstrated that in the two brain regions, including Nac and subic, 14 and 16 genes, respectively, were found to reach tissue- enhanced level, that is, these two brain areas play a critical role in the development of depression (Table 2; Supplementary Table 7). The gene *MYO3A* encoding myosin IIIA, was highly enriched in the Nac tissues of all three groups, which suggested that *MYO3A* gene as Nac tissue specific gene is unrelated to the phenotype. At the same time, the expression of *TNFRSF8* gene was enhanced into the Nac of the control group and the group of suicide with MDD, but not in the group of suicide without MDD. Another gene, *TARID transcript* as a non-coding RNA, was found in the Nac of control and suicide without MDD, but not in the suicide with MDD. We speculated that *TNFRSF8* and *TARID* were considered as not only the tissue specific genes but also phenotype-specific genes. For the subic tissue, no genes as tissue-enhanced genes were shared among three groups. In addition, *CLIC6*, encoding chloride intracellular channel 6, enriched in the subic of both suicide without MDD and control, but was absent in the suicide with MDD.

**Table 2:** The enhanced level genes from *TissueEnrich* analysis distributed into the groups and tissues.

Group	Gene name	Description	Biotype	Enhanced tissues	p value
CON	MCOLN3	Mucolipin TRP cation channel 3	Protein_coding	Subic	2.84E−08
	**TARID**	**TARID**	**Antisense**	**Nac**	**0.00003**
	F5	Coagulation factor V	Protein_coding	Subic	0.0150
	**MYO3A**	**Myosin IIIA**	**Protein_coding**	**Nac**	**0.0009**
	MORC1	MORC family CW-type zinc finger 1	Protein_coding	Subic	0.0014
	SLC16A12	Solute carrier family 16 member 12	Protein_coding	Subic	0.0505
	**TNFRSF8**	**TNF receptor superfamily member 8**	**Protein_coding**	**Nac**	**0.0034**
	CYP4F32P	Unprocessed pseudogene	Pseudogene	Nac	0.0136
	LINC00534	Long intergenic non-protein coding RNA 534	LincRNA	Nac	0.0108
	**CLIC6**	**Chloride intracellular channel 6**	**Protein_coding**	**Subic**	**0.0189**
	RP1-283K11.2	RP1-283K11.2	Antisense	Nac	0.0010
	RP11-343J3.2	RP11-343J3.2	Antisense	Subic	0.0009
	SHISA6	Shisa family member 6	Protein_coding	Subic	0.0020
	SHOX2	Short stature homeobox 2	Protein_coding	Subic	0.1292
	TSPAN18	Tetraspanin 18	Protein_coding	Subic	0.0303
	CP	Ceruloplasmin	Protein_coding	Subic	0.0558
	SLC5A5	Solute carrier family 5 member 5	Protein_coding	Subic	0.0774
S−D	ABCA12	ATP binding cassette subfamily A member 12	Protein_coding	Subic	0.000002
	**TARID**	**TARID**	**Antisense**	**Nac**	**0.0033**
	GUCA1C	Guanylate cyclase activator 1C	Protein_coding	Subic	0.0030
	RPL36AP53	Ribosomal protein L36a pseudogene 53	Pseudogene	Nac	0.0003
	RP11-60A8.1	RP11-60A8.1	LincRNA	Subic	0.00007
	GPR1	G protein-coupled receptor 1	Protein_coding	Nac	0.0006
	**MYO3A**	**Myosin IIIA**	**Protein_coding**	**Nac**	**0.0017**
	AMBN	Ameloblastin	Protein_coding	Nac	0.0037
	UPK1B	Uroplakin 1B	Protein_coding	Nac	0.0028
	LINC00645	Long intergenic non-protein coding RNA 645	LincRNA	Nac	0.0263
	RXFP2	Relaxin family peptide receptor 2	Protein_coding	Nac	0.0005
	CTB-32P11.1	Uncharacterized LOC101927862	LincRNA	Subic	0.00009
	LL21NC02-1C16.2	LL21NC02-1C16.2	Antisense	Nac	0.0030
	**CLIC6**	**Chloride intracellular channel 6**	**Protein_coding**	**Subic**	**0.0051**
S+D	SKAP1	src kinase associated phosphoprotein 1	Protein_coding	Subic	1.10E−12
	**MYO3A**	**Myosin IIIA**	**Protein_coding**	**Nac**	**0.0000012**
	**TNFRSF8**	**TNF receptor superfamily member 8**	**Protein_coding**	**Nac**	**0.0000079**
	RP11-478C6.6	Novel zinc finger protein pseudogene	Pseudogene	Nac	0.0000271

The bold font characters mean significant genes.

### Sex specific signatures of expression patterns in suicides

By means of cross phenotype tissue analysis, we calculated the R and p value of all transcript genes for the total subject group, male group, and female group. We also ranked the statistical R value of all significant transcripts and listed the top genes in Table 3. The top R genes in the mixed gender group (*PRS26* (R = 0.37), *ARNT* (R = 0.32) and *SYN3* (R = 0.32), the male group (*FBXO7* (R = 0.41), *RNU6-951P* (R = 0.40) and *ACTR8* (R = 0.39)) and the female group (*ADI1* (R = 0.64), *CH25H* (R = 0.63) and *CCR1* (R = 0.57), were identified as the most differentially expressed genes across two variables of tissues and phenotypes (Table 3; Supplementary Fig. 7). To identify differences between male and female in global expression patterns across three phenotypes and multiple tissues, the results of rank–rank hypergeometric overlay analysis showed that there was no significant overlap of global gene expression patterns were detected between male and female groups (p value = 0.92) (Supplementary Fig. 8).

**Table 3:** The top Significant genes with high statistic value by cross-phenotype-tissue analysis.

Group	Ensemble_ID	Gene_symbol	Description	Biotype	Statistic R	Significance
Mixed	ENSG00000197728	RPS26	Ribosomal protein S26	Protein_coding	0.371	0.001
	ENSG00000143437	ARNT	Aryl hydrocarbon receptor nuclear translocator	Protein_coding	0.326	0.001
	ENSG00000185666	SYN3	Synapsin III	Protein_coding	0.324	0.001
	ENSG00000259675	RP11-507B12.1	Novel transcript	LincRNA	0.317	0.001
	ENSG00000233273	AMMECR1LP1	Pseudogene	Pseudogene	0.316	0.001
	ENSG00000174456	C12orf76	Chromosome 12 open reading frame 76	Protein_coding	0.315	0.001
	ENSG00000258711	RP11-218E20.3	Novel transcript	LincRNA	0.313	0.001
	ENSG00000138135	CH25H	Cholesterol 25-hydroxylase	Protein_coding	0.313	0.001
	ENSG00000200832	SNORD114-4	Small nucleolar RNA, C/D box 114–4	snoRNA	0.307	0.001
	ENSG00000102878	HSF4	Heat shock transcription factor 4	Protein_coding	0.306	0.001
Male	ENSG00000100225	FBXO7	F-box protein 7	Protein_coding	0.406	0.001
	ENSG00000199603	RNU6-951P	RNA, U6 small nuclear 951, pseudogene	snRNA	0.400	0.001
	ENSG00000113812	ACTR8	ARP8 actin-related protein 8 homolog (yeast)	Protein_coding	0.387	0.001
	ENSG00000252035	RNU6-397P	RNA, U6 small nuclear 397, pseudogene	snRNA	0.387	0.001
	ENSG00000212100	MIR764	MicroRNA 764	MiRNA	0.375	0.001
	ENSG00000231713	AF064860.7	LincRNA	LincRNA	0.361	0.001
	ENSG00000226684	RP3-418C23.2	LincRNA	LincRNA	0.331	0.001
	ENSG00000106069	CHN2	Chimerin 2	Protein_coding	0.330	0.001
	ENSG00000233273	AMMECR1LP1	Pseudogene	Pseudogene	0.328	0.001
	ENSG00000162645	GBP2	Guanylate binding protein 2, interferon-inducible	Protein_coding	0.308	0.001
Female	ENSG00000182551	ADI1	Acireductone dioxygenase 1	Protein_coding	0.640	0.001
	ENSG00000138135	CH25H	Cholesterol 25-hydroxylase	Protein_coding	0.631	0.001
	ENSG00000163823	CCR1	C–C motif chemokine receptor 1	Protein_coding	0.570	0.001
	ENSG00000141469	SLC14A1	Solute carrier family 14 member 1	Protein_coding	0.559	0.001
	ENSG00000230870	FBXW11P1	Pseudogene	Pseudogene	0.548	0.001
	ENSG00000214198	RP11-642P15.1	Pseudogene	Pseudogene	0.548	0.001
	ENSG00000256943	RP13-895J2.2	Antisense	Antisense	0.548	0.001
	ENSG00000232640	RP1-266L20.2	Antisense	Antisense	0.546	0.001
	ENSG00000129480	DTD2	d-Tyrosyl-tRNA deacylase 2 (putative)	Protein_coding	0.544	0.001
	ENSG00000206712	RNU6-26P	RNA, U6 small nuclear 26, pseudogene	snRNA	0.541	0.001

## Discussion

In this study, we identified some novel targets that can be used to distinguish different types of postmortem brain tissues from depression with or without suicide and suicide without depression. In addition, we also examined the tissue-specific and sex-specific transcriptional patterns between suicide dependent or independent of depression. Here, we selected two classical genome wide expression datasets of postmortem brain tissues from depression or suicide and extracted the demographic information of human postmortem brain tissue about having suicide or non-suicide, from the perspective of commodity phenotypes or single phenotype to find the possible key genes or co-expression patterns. Considering differentially expressed genes may correlate with different phenotypes, brain tissues and sex, various statistical strategies including ANOVA, *limma* and Vegan were adapted to conduct WGCNA, machine learning and RROH. We found that most of the DEGs, especially the top significant genes, were consistent with previous studies, such as *MRPS6, SERPINH1*and *IL8* gene in GSE101521, *DUSP6*, *EMX1* and *MPAK15* gene in GSE102556. In addition, we also identified novel possible transcripts and ncRNA that contribute to the discrimination of depression or suicide.

Postmortem brain tissue as an important material reflects the molecular and cellular changes in brain tissues from suicided victims and controls, which are also features of mental illness^11^. However, the prominent consideration of postmortem brain studies is the impact of comorbid suicide diagnoses including some common psychiatric disorders and other risk factors that influence the transitions from suicide ideation to suicide attempts^61^. From the perspective of suicide with psychiatric disorders, Underwood et al.^62^ investigated brain serotonin transporter, 5-HT1A and 5-HT2A receptor binding in the prefrontal cortex and anterior cingulate cortex of suicide and controls. They reported that lower serotonin transporter levels in suicide were dependent on MDD, and found that higher 5-HT1A binding in suicides was independent of MDD, whereas 5-HT2A binding increased in suicides with MDD or alcoholism. This study suggested serotonin receptor binding in postmortem brain tissue can be a risk factor to separate the effects of suicide from comorbid MDD, AUD, and early life adversity^62^. In the present study, we explored the suicide- or depression-specific mRNA and mRNA co-expression pattern changes that contribute to development of suicide and depression. From the WGCNA results, the biological functions of co-expression pattern associated with suicide without depression are mainly related to ncRNA processing activity, whereas depression without suicide can be attributed to neuronal synapses, suggesting that epigenetic modification and synaptic dysfunction in brain tissues play an essential role in the development of suicide or depression.

For machine learning analysis, we integrated contrast analyses of multiple comparisons and machine learning to identify candidate targets for a given sub-phenotype. In this process, we first maximally optimized the various parameters for building the training model by means of *randomforest* and obtained relatively accurate distinguishing capacity. To confirm the distinguishing accuracy of genes with high MeanDecreaseGini, we compared the AUC score between *randomforest* models of genes with high MeanDecreaseGini and the group of all genes, the results of which shown AUC score of genes with high MeanDecreaseGini were largely enhanced in the S + D_1 (AUC _high MeanDecreaseGini genes_ = 0.90; AUC _total genes_ = 0.81). Intriguingly, we also found majority of these genes with high MeanDecreaseGini belong to the family of non-coding RNAs. Moreover, several ncRNAs including *GCNT1P1*, *AC092745.1*, *ASH1L-IT1* and *RNU6-581P* from the S + D_1 group are specifically enriched in the cerebellum and pituitary by the analysis of gene expression levels across different human tissues in the GTEx dataset, suggesting that ncRNAs that are highly expressed in the cerebellum and pituitary may be used as specific targets for suicide and MDD^63^. Currently, machine learning analysis are used for diagnosis, prognosis, treatment decisions, and detection of biomarker for suicide^64,65^. To predict suicide attempts and suicide deaths, the prediction model was developed using the penalized LASSO method with health record data and self-report questionnaires from about 3 million patients, and the results have shown an accuracy ranging between 0.83 and 0.85^66,67^. Meanwhile, machine learning was used for exploring biomarkers of the antidepressant by analyzing the expression of microRNA in previous MDD studies^68^.

*TissueEnrich* was used to find out the essential tissues for the development of depression and identify tissue-specific genes, and co-expression patterns associated with tissues. Interestingly, the enhanced genes in the *TissueEnrich* suggested that the Nac region may be the most important tissue contributing to the pathophysiology and symptomatology of depression and suicide. Under normal conditions, Nac function is related to the brain’s limbic and reward circuits and the uptake of glutamatergic and dopaminergic signals from other brain tissues^69^. Evidence from the rodent depression model and the antidepressant-like effects also support that Nac tissues are involved in the impaired motivation, anhedonia, and decreased energy levels which are core symptoms of depression^70^. Despite that genome-wide transcriptional study supports that several molecular changes in the Nac of depressive like behavior animal models, we further validated the importance of Nac and subic for suicide or depression^71^.

We have uncovered the global landscape of distinct expression patterns for different tissues and individuals in depression and suicide. In recent years, the development of multi-dimension informatics approaches including *vegan* and *Tensorflow*, has enabled the global investigation of significantly dysregulated gene expression across multiple variables, and have identified key genes for the concomitant diseases. The subsequent RRHO analysis^58^ further confirmed that the global gene expression patterns have little overlap between the male and female group under the situations of suicide independent depression or suicide comorbid depression, which is consistent with the conclusions of previous transcriptomic studies in depression^43,59,72,73^.

At present, transcriptional studies from candidate genes to genome-wide approaches have also identified some dysregulated genes associated with sex difference in MDD. Some candidate genes, from serotonergic system to GABA synthesizing enzymes, were found to have altered expression associated with sex difference in MDD ^72^. Particularly some key genes identified from transcriptomic studies, e.g., DUSP6, EMX1, LINC00473, as potential important drivers for sex difference, directly involved in the brain molecular changes in depressed men and women^43^. Additionally, novel approaches including global analysis in present study and gene co-expression network analysis and deconvolution analyses provided more convincing evidence that the existence of sex differences across the brain in the MDD. About the underlying mechanisms on sex difference at the behavioral or transcriptional levels, evidence has suggested that the interplay between sex chromosomes, development gonadal hormones and adult hormone exposure could influence social behavior, alcohol abuse, habit formation, aggressive and parenting behavior, and gene expression, which could mediate the different mood changes in male and female MDD patients^72,74^.

In summary, we investigated different transcriptomic datasets to uncover differential gene targets and pathways among subgroups of depression with suicide, suicide without depression and depression without suicide. We also investigated the co-expression patterns and hub genes significantly associated with these three sub-phenotypes. Meanwhile, global analysis across three phenotypes and tissues confirmed evidence for sex difference in mood disorders. Our study is still limited by the sample size in each subgroup, as well as limited detailed clinical information and medical history on postmortem brain tissues.

## URLs

1. Heatmap: https://cran.r-project.org/web/packages/gplots/index.html.
2. WGCNA analysis: https://horvath.genetics.ucla.edu/html/CoexpressionNetwork/Rpackages/WGCNA/Tutorials/.
3. Machine learning analysis Rando: https://topepo.github.io/caret/model-training-and-tuning.html, https://rpubs.com/phamdinhkhanh/389752.
4. TissueEnrich analysis: https://bioconductor.org/packages/release/bioc/vignettes/TissueEnrich/inst/doc/TissueEnrich.html.
5. Vegan analysis: https://cran.r-project.org/web/packages/vegan/vegan.pdf.
6. RRHO analysis: https://systems.crump.ucla.edu/rankrank/rankranksimple.php.

## Supplementary Information

Supplementary Table 1.

Supplementary Table 2.

Supplementary Table 3.

Supplementary Table 4.

Supplementary Table 5.

Supplementary Table 6.

Supplementary Table 7.

Supplementary Information.

## Data Availability

The authors declare that all the data supporting the findings of this study are available from the GEO database (GSE102556 and GSE101521); and some secondary results can be found in its supplementary information files.

## Code availability

The authors declare that all the codes supporting the findings of this study are available from the corresponding author on reasonable request.
