# Supplementary material for: Identification of novel targets and pathways to distinguish suicide dependent or independent on depression diagnosis
Source: Sci Rep. 2023 Feb 13;13:2488. doi: 10.1038/s41598-023-29101-1 (PMC9925752; doi:10.1038/s41598-023-29101-1)
Supplement: Supplementary file 8 — Supplementary Information. [file 41598_2023_29101_MOESM8_ESM.docx]

**Identification of novel targets and pathways to distinguish suicide dependent or independent on depression diagnosis**

Siqi Peng ^1^, Yalan Zhou ^1^, Lan Xiong^2^**^#^**, Qingzhong Wang ^1#^

**1** Institute of Chinese Materia Medica, Shanghai University of Traditional Chinese Medicine, Shanghai 201203, China

2. Montreal Neurological Institute and Hospital, McGill University, Montreal, QC, Canada.

### Contributions

Qingzhong Wang, Lan Xiong designed the project, checked and edited the manuscript. Material preparation and data collection and analysis were performed by Siqi Peng and Yalan Zhou. All authors have read and approved the final version of the manuscript.

**# Corresponding authors:** Qingzhong Wang and Lan Xiong

Qingzhong Wang: [wangqingzhong3@gmail.com](mailto:wangqingzhong3@gmail.com); [qzwang2018@sibs.ac.cn](mailto:qzwang2018@sibs.ac.cn)

Lan Xiong: lan.xiong@mcgill.ca


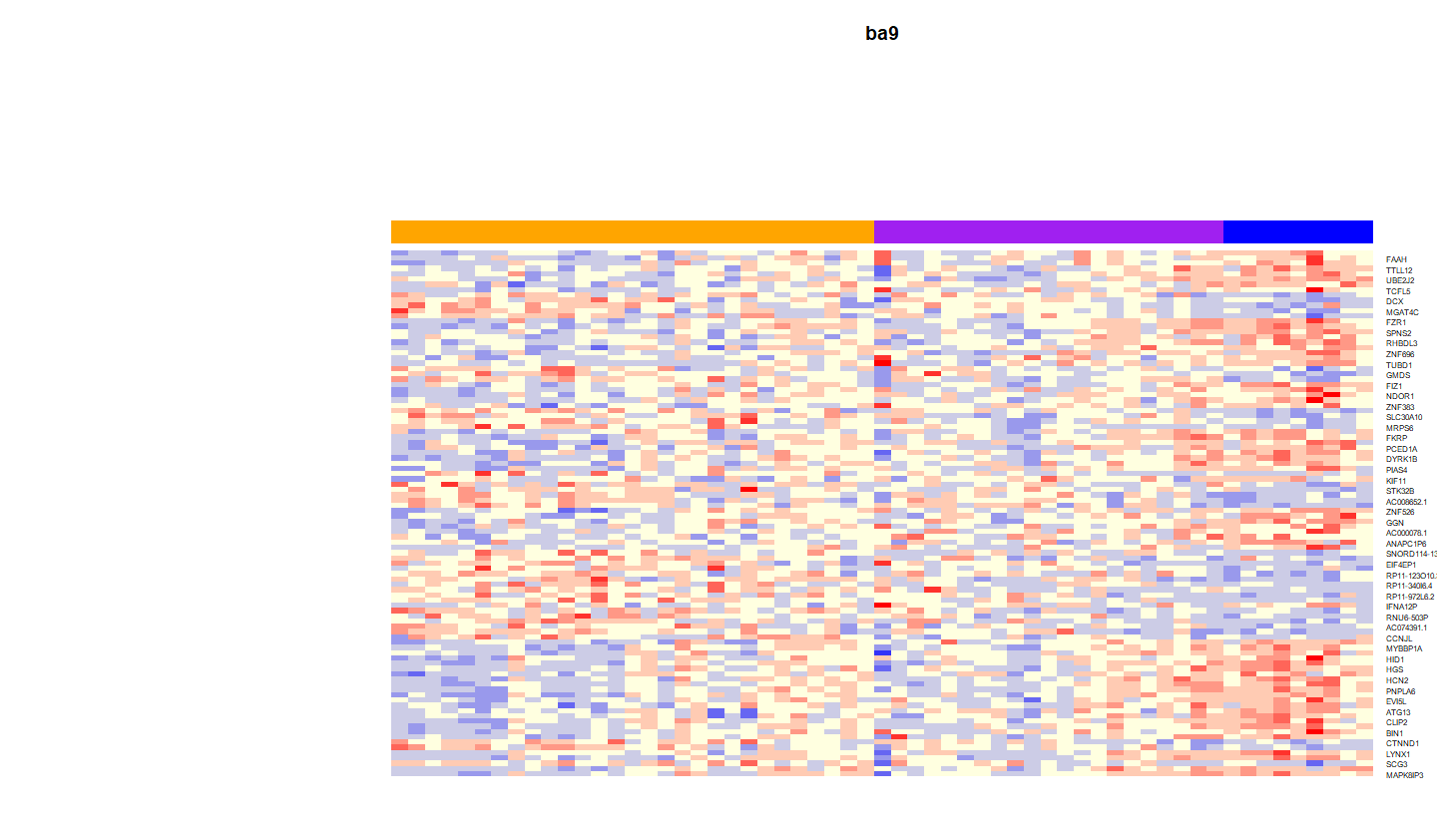


**Supplementary Figure 1.** The most significant difference in expression level across three Depression with suicide, depression without suicide and healthy controls. The top altered genes expression from cross-phenotype analysis in the Suicide Group. The heatmap plot were conducted by the heatmap.2 fuction in the gplots package (v3.1.3).

**2A**

**2B**


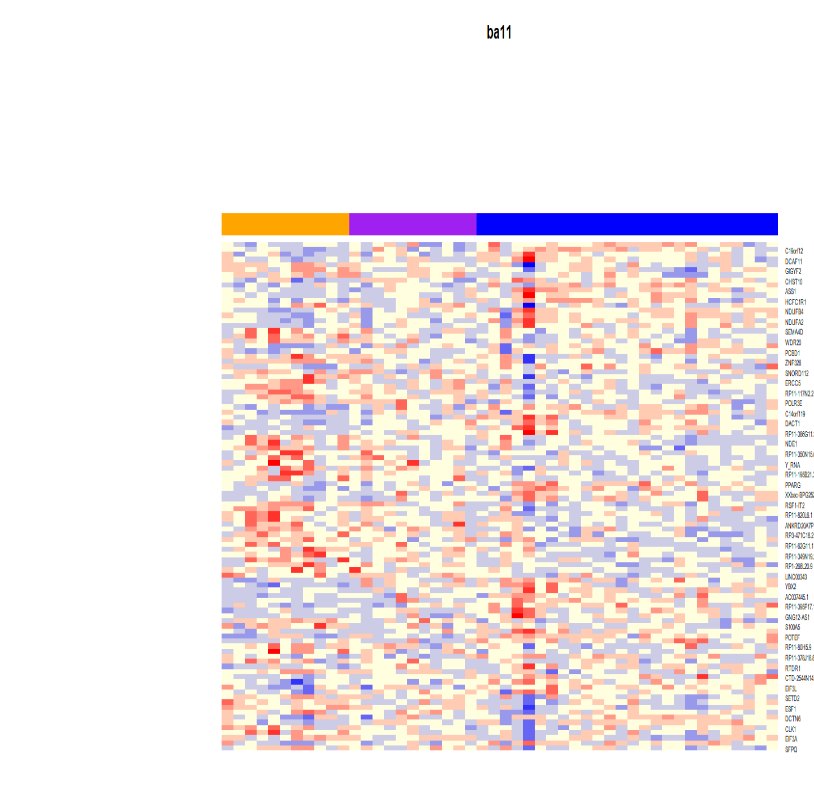


**2C**

**2D**

**2E**

**2F**

**Supplementary Figure 2.** Heatmap of the top dysregulated genes that are differentially expressed across three groups by cross-phenotype analysis (Tissue type number =6). A. The top significant gene expression changes in the anterior Insula area(ANT). B. Orbitofrontal (BA 11) of Suicide Group. C. Cingulate gyrus 25 (BA25) of Suicide Group. D. Dorsolateral prefrontal cortex (BA8/9) of Suicide Group. E. Nucleus Accumbens (NAC) of Suicide Group. F. Subiculum (Subic) of Suicide Group. The heatmap figure were plotted by the heatmap.2 fuction in the gplots package (v3.1.3).


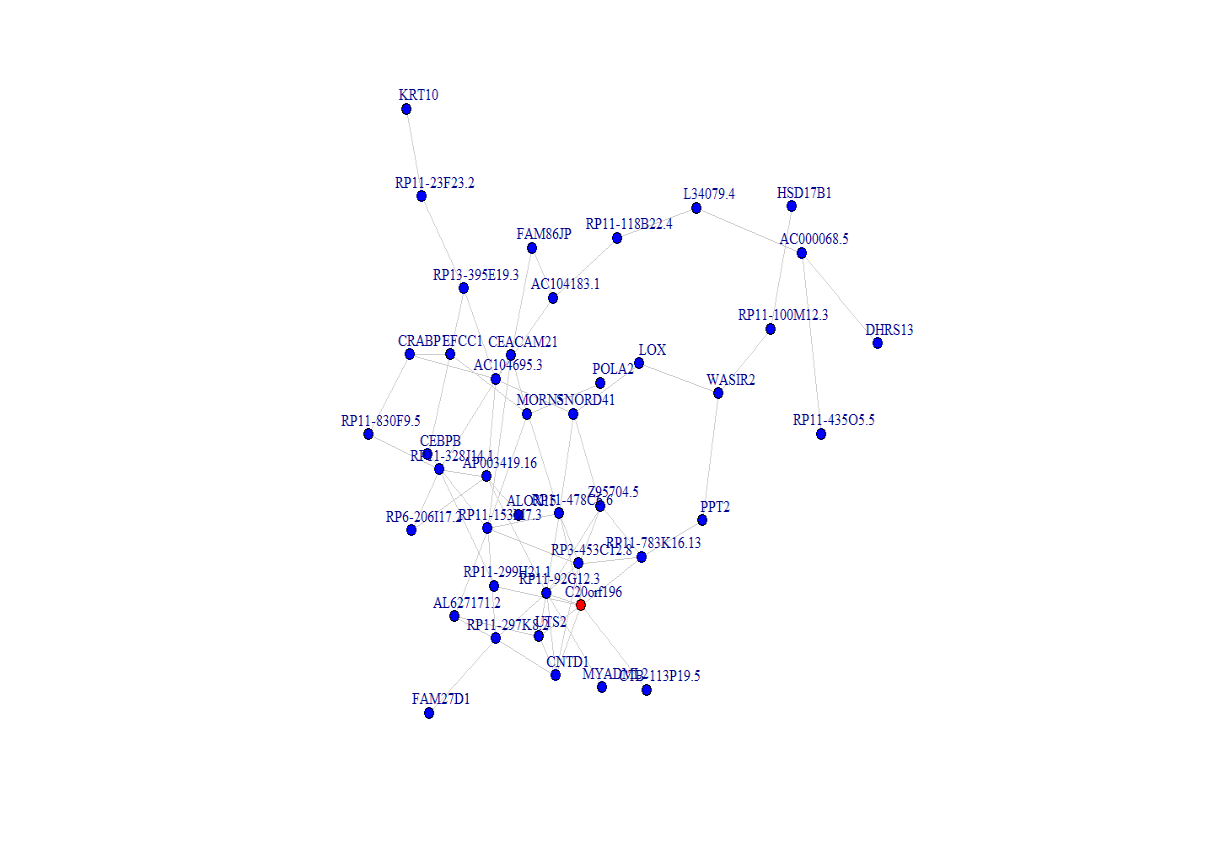


Figure 3A


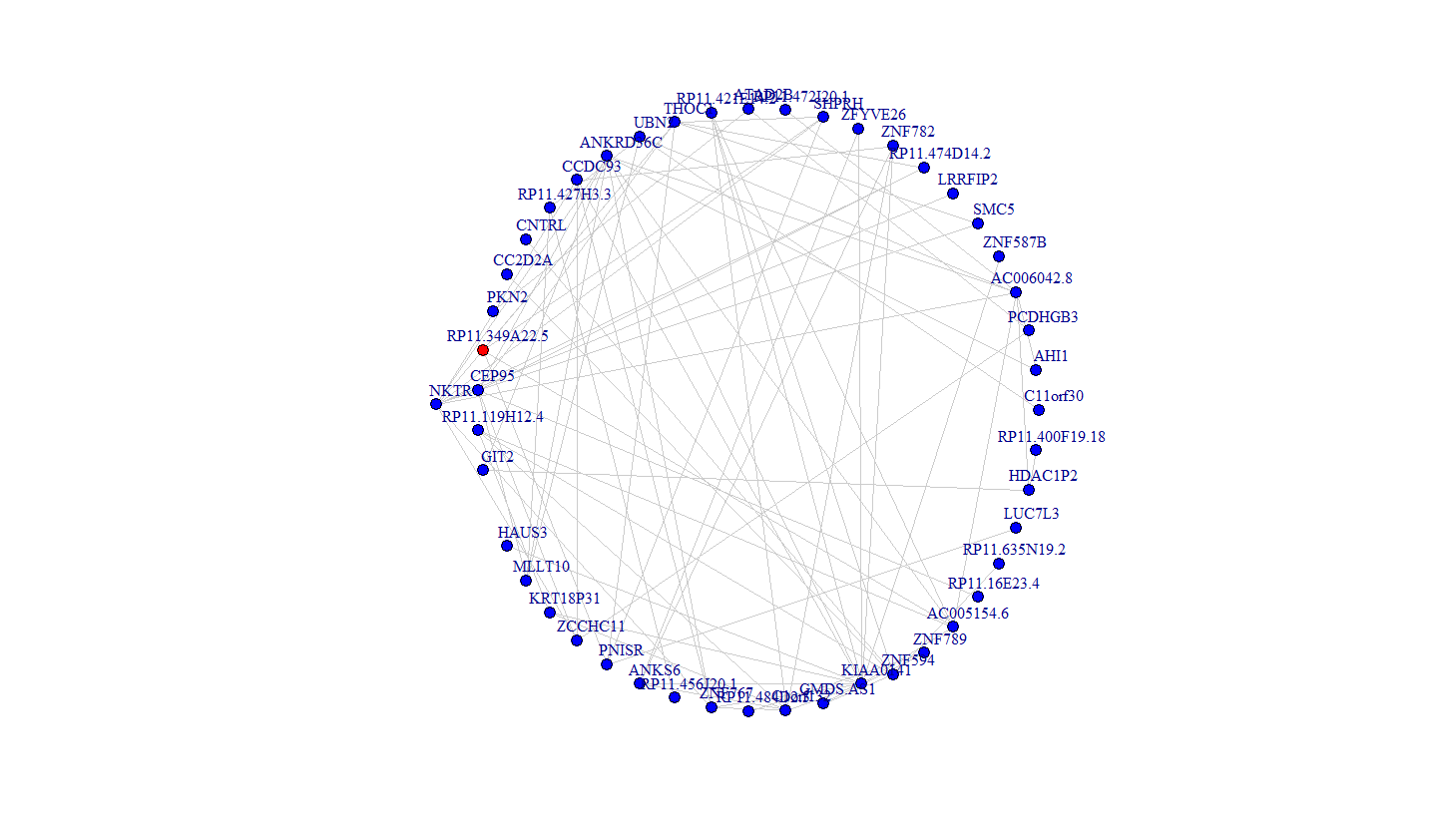


Figure 3B


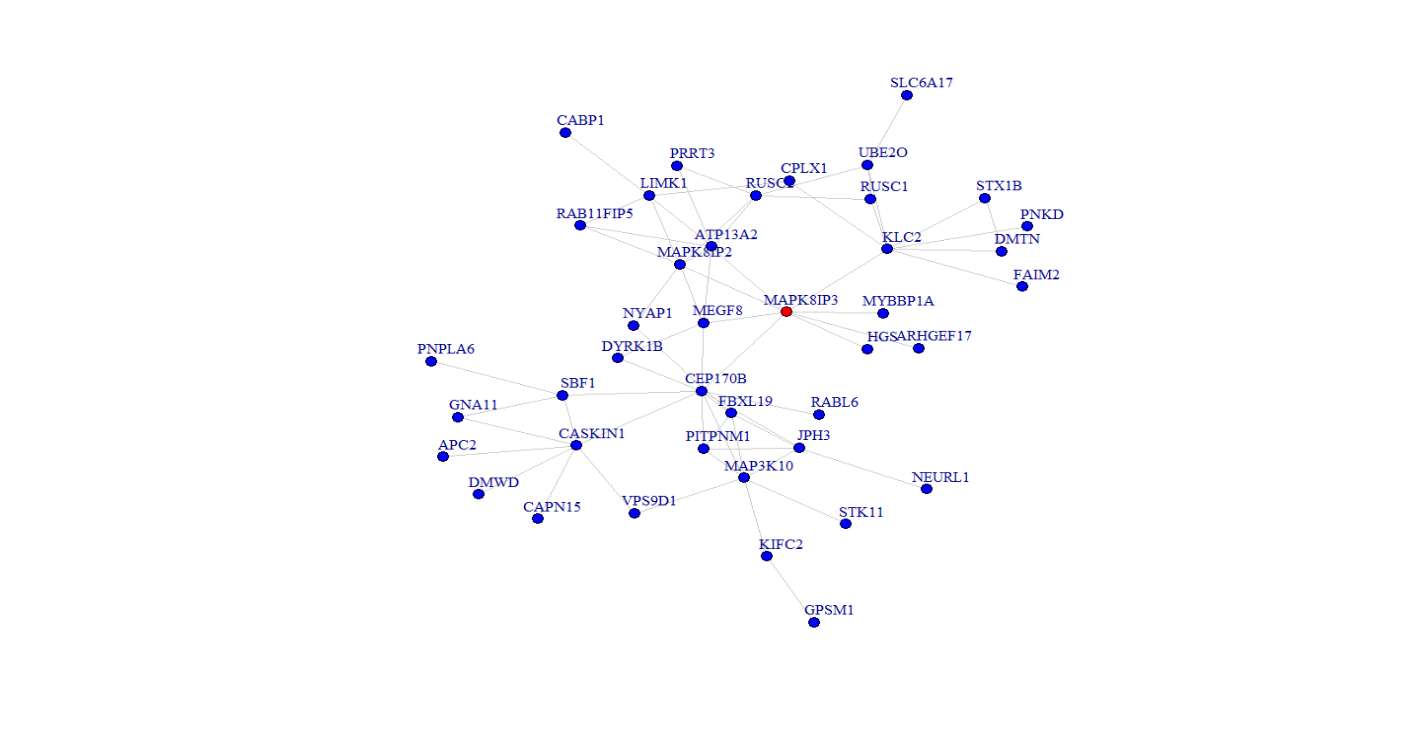


Figure 3C


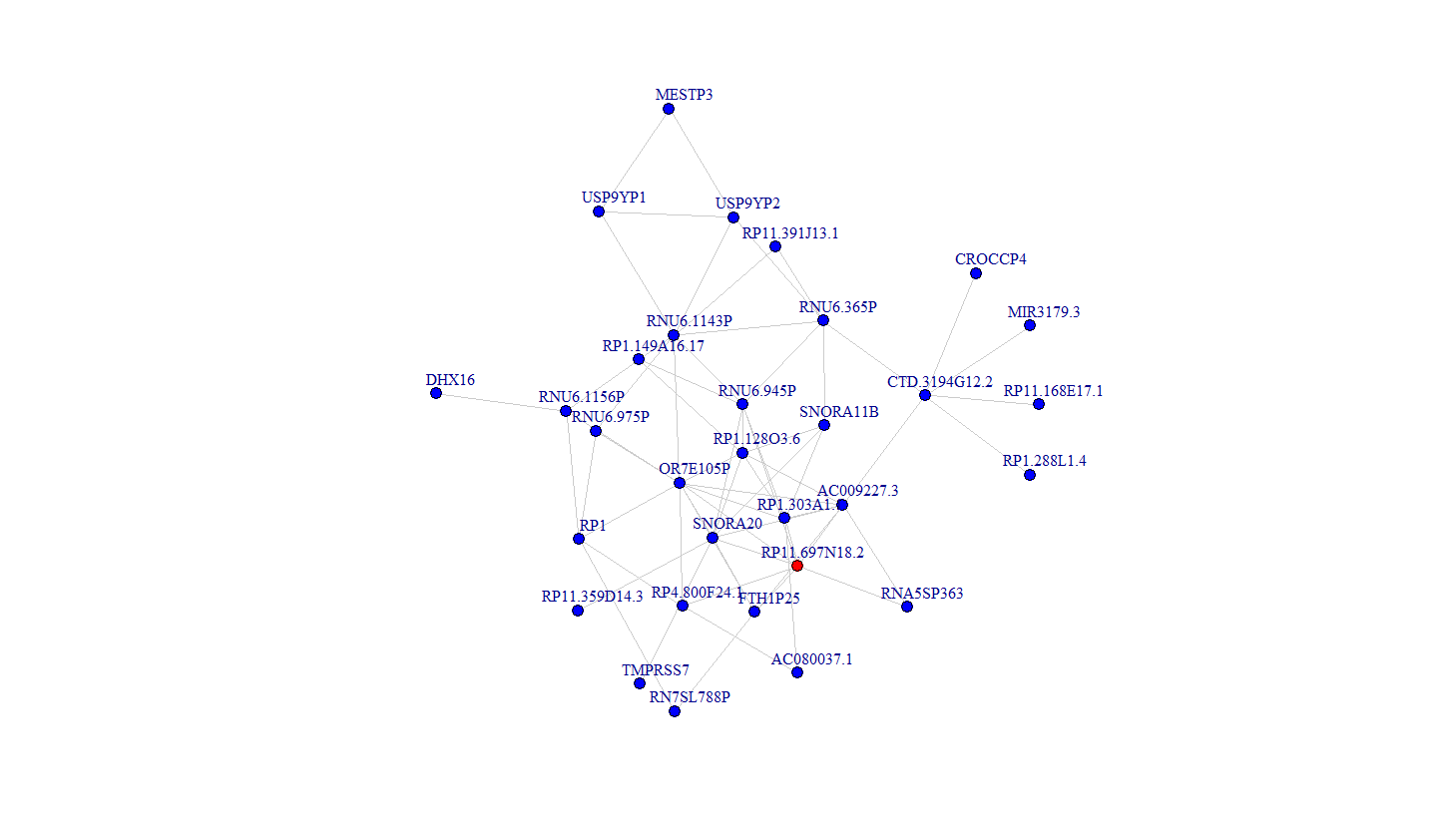


Figure 3D

**Supplementary Figure 3.** Subset of the most connected genes (intra-modular connectivity >0.9) in each associated-phenotypic modules. Red dots represent the hub genes. a. *C20orf196* gene in the midnightblue module. b. *RP11-349A22.5* gene in the blue module. c. *RP11-697N18.2* gene in the orange module. d. *MAPK8IP3* gene in the pink module.


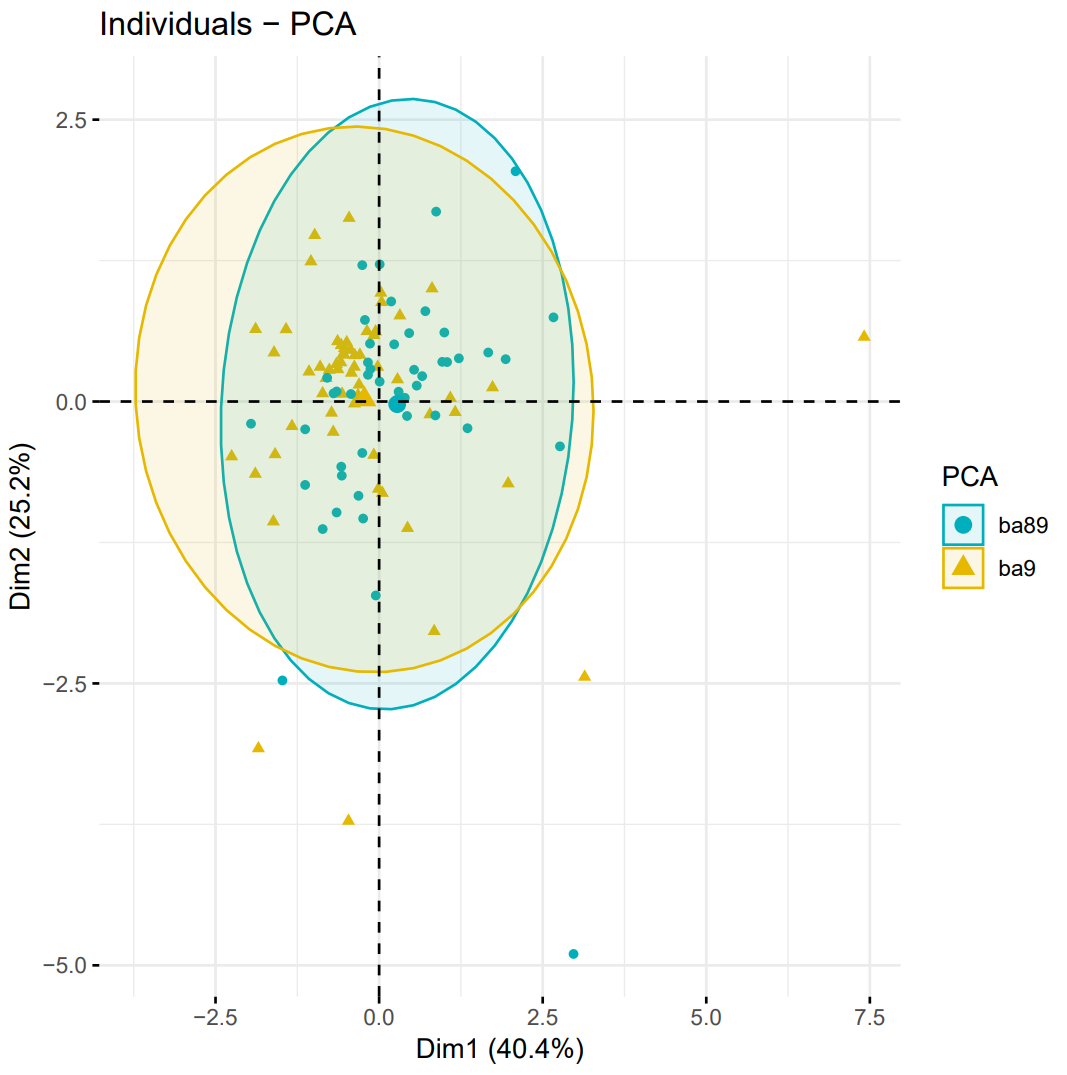


Frontal cortex in the Suicide Group

Frontal cortex in the Depression Group

**Supplementary Figure 4.** PCA two-dimensional scatter plots represent the differential gene expression patterns of the different human frontal cortex from two groups. Each dot represents a sample and the color its origin: green dots represent frontal cortex of Suicide Group; yellow dots represent frontal cortex of Depression Group.

**5A**

**5B**

**5C**

**Supplementary Figure 5.** Variable importance as measured by mean decrease Gini index using *RandomForest* model. A mean decrease in Gini index indicates median importance and better classification for the disease state with the selected DEGs. Figure 2A. the group of suicide with MDD; Figure 2B. Suicide without MDD; Figure 2C: MDD without suicide.

**6A**

**6B**

**Supplementary Figure 6**. The heatmap illustrates the top DGEs expression pattern from the cross-tissue analysis in the three phenotypes groups.

Figure 3A. The heatmap of the significant genes expression profiling in the suicide with MDD. Figure 3B. the heatmap of the suicide without MDD. Figure 3C. the heatmap of healthy control. These figures was performed with the heatmap.2 fuction in the gplots package (v3.1.3).


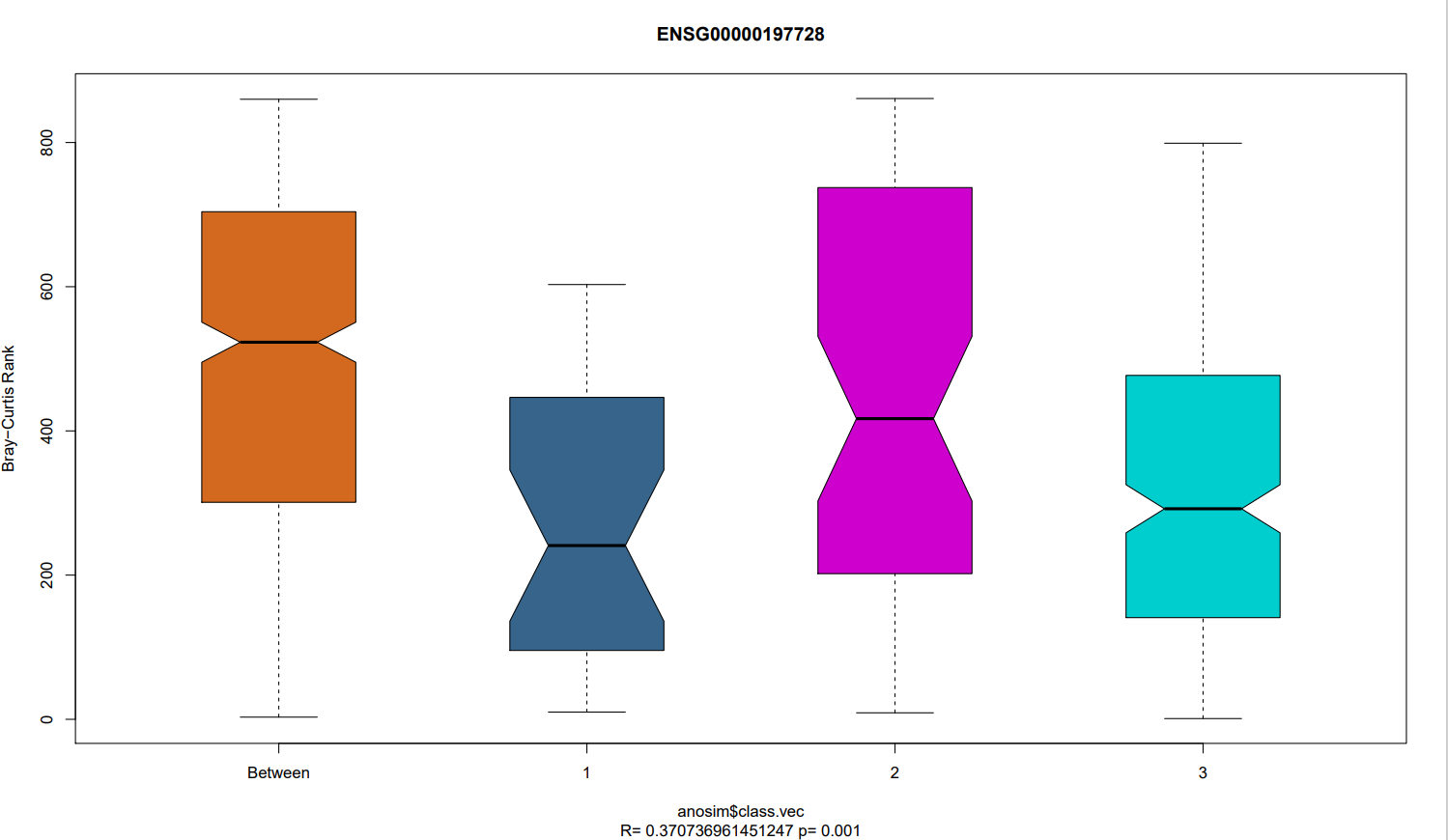

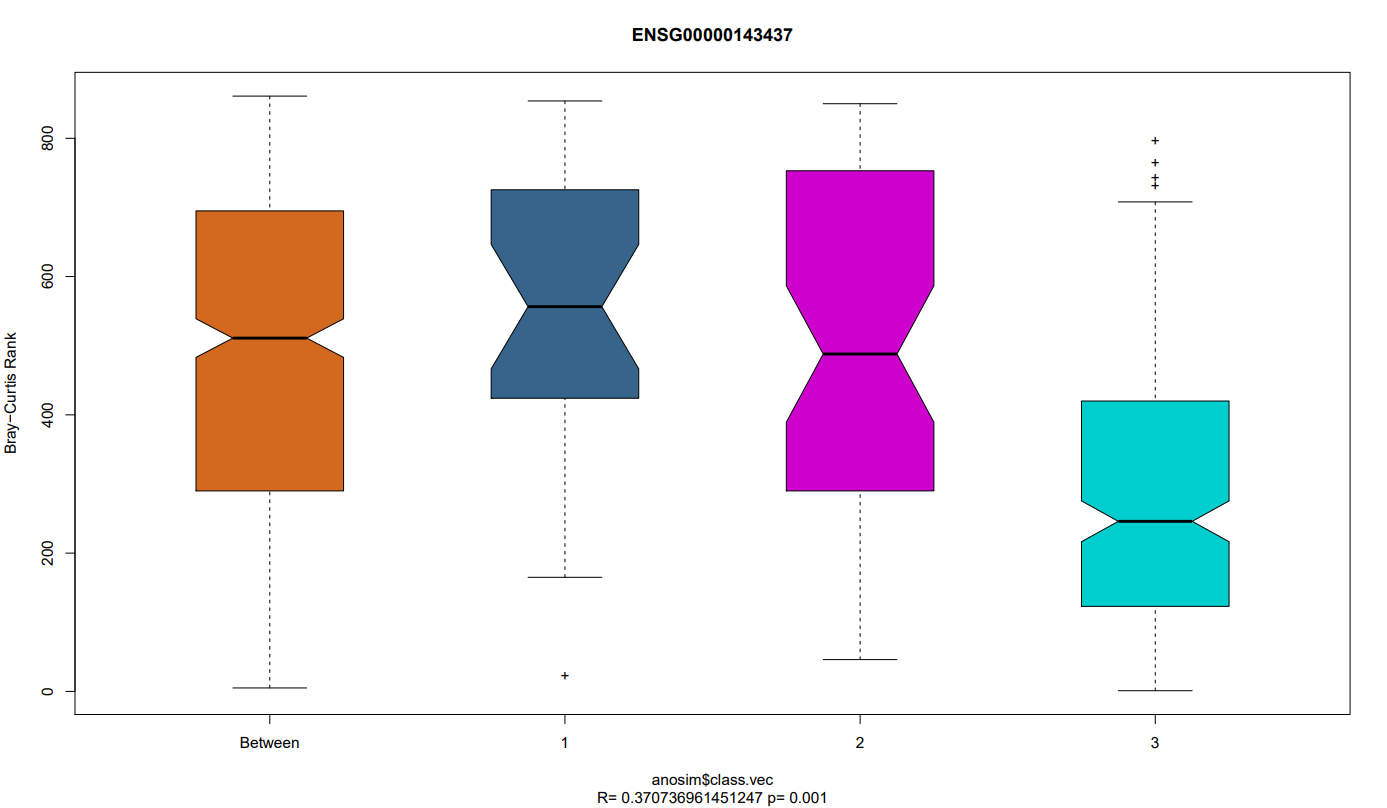


RPS26

ANRT


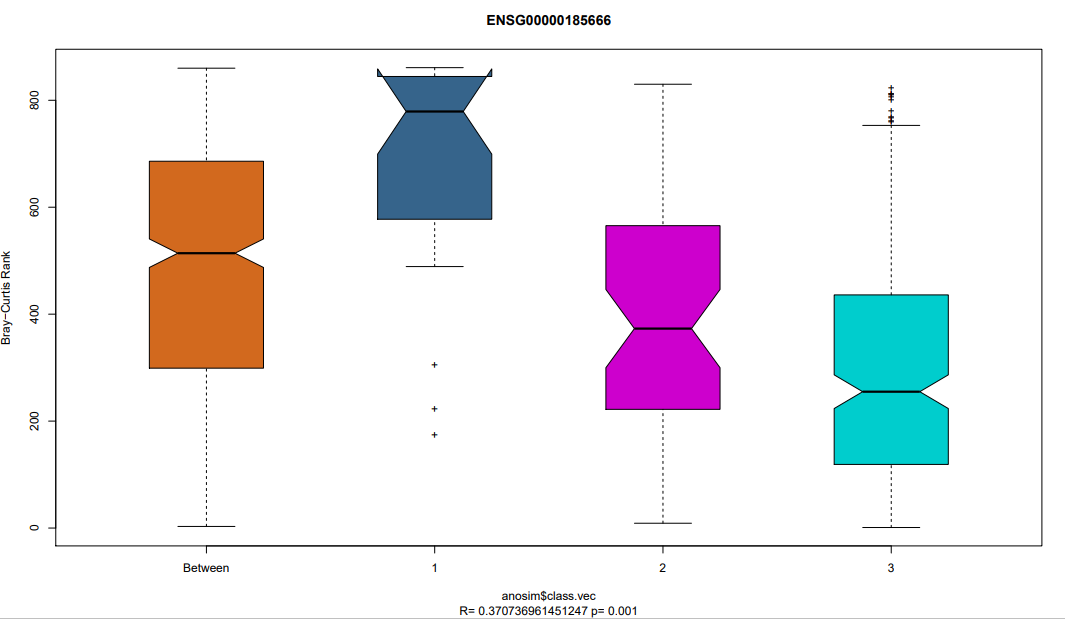


SYN3

Between

CON

S-M

S+M

Between

CON

S-M

S+M

Between

CON

S-M

S+M

**Supplementary Figure 7.** Analysis of similarities (ANOSIM) plot of the three top genes by cross-disease-tissue analysis, which have shown dissimilarity between and within groups. Figure 4A. *RPS26* gene (R = 0.37, p= 0.001); Figure 4B. *ARNT* (R= 0.32, p-value = 0.001); Figure 4C. *SYN3* (R= 0.32, p-value = 0.001).

male group

Female group

**Supplementary Figure 8.** Rank-rank hypergeometric overlap (RRHO) maps compare differential expression between males and females (male, row panel; female, column panel).
